# A simulation study on the impact of the blood flow-dependent component in [^18^F]AV45 SUVR in Alzheimer’s disease

**DOI:** 10.1371/journal.pone.0189155

**Published:** 2017-12-06

**Authors:** Julie Ottoy, Jeroen Verhaeghe, Ellis Niemantsverdriet, Sebastiaan Engelborghs, Sigrid Stroobants, Steven Staelens

**Affiliations:** 1 Molecular Imaging Center Antwerp, University of Antwerp, Antwerp, Belgium; 2 Reference Center for Biological Markers of Dementia (BIODEM), University of Antwerp, Antwerp, Belgium; 3 Department of Neurology and Memory Clinic, Hospital Network Antwerp (ZNA) Hoge Beuken en Middelheim, Antwerp, Belgium; 4 Department of Nuclear Medicine, Antwerp University Hospital, Edegem, Belgium; Wayne State University, UNITED STATES

## Abstract

**Background:**

Increased brain uptake on [^18^F]AV45 PET is a biomarker for Alzheimer’s disease (AD). The standardised uptake value ratio (SUVR) is widely used for quantification but is subject to variability. Here we evaluate how SUVR of a cortical target region is affected by blood flow changes in the target and two frequently used reference regions.

**Methods:**

Regional baseline time-activity curves (TACs) were simulated based on metabolite-corrected plasma input functions and pharmacokinetic parameters obtained from our previously acquired data in healthy control (HC; n = 10), amnestic mild cognitive impairment (aMCI; n = 15) and AD cohorts (n = 9). Blood flow changes were simulated by altering the regional tracer delivery rate K1 (and clearance rate k2) between -40% and +40% from its regional baseline value in the target region and/or cerebellar grey (CB) or subcortical white matter (WM) reference regions. The corresponding change in SUVR was calculated at 50–60 min post-injection.

**Results:**

A -40% blood flow reduction in the target resulted in an increased SUVR^target^ (e.g. SUVR^precuneus^: +10.0±5% in HC, +2.5±2% in AD), irrespective of the used reference region. A -40% blood flow reduction in the WM reference region increased SUVR_WM_ (+11.5±4% in HC, +13.5±3% in AD) while a blood flow reduction in CB decreased SUVR_CB_ (-9.5±6% in HC, -5.5±2% in AD), irrespective of the used target region. A -40% flow reduction in both the precuneus and reference WM (i.e., global flow change) induced an increased SUVR (+22.5±8% in HC, +16.0±4% in AD). When considering reference CB instead, SUVR was decreased by less than -5% (both in HC and AD).

**Conclusion:**

Blood flow changes introduce alterations in [^18^F]AV45 PET SUVR. Flow reductions in the CB and WM reference regions resulted in a decreased and increased SUVR of the target, respectively. SUVR was more affected by global blood flow changes when considering WM instead of CB normalization.

## Introduction

Alzheimer’s disease (AD), the most common cause of dementia, is associated with excessive accumulation of amyloid-β (Aβ) peptides and hyperphosphorylated tau in the brain [1]. These proteins form amyloid plaques and neurofibrillary tangles that cause pathological changes in the brain, leading to synaptic loss, neuronal cell death and cognitive deterioration [2,3]. As cortical amyloid deposition is one of the earliest markers for AD pathology, amyloid PET imaging is a valuable tool for early detection and direct quantification of Aβ plaque load in the brain. The second generation amyloid PET-tracer [^18^F]AV45 ([^18^F]florbetapir commercial name Amyvid™, Eli Lilly [4]) recently became FDA- and EMA-approved in patients due to its high selectivity for Aβ plaques, fast kinetics and the long physical half-life of ^18^F (110 min) [5].

The most widely used quantification index in amyloid PET is the standardised uptake value ratio (SUVR), which is the ratio of radioactivity concentration of the target and reference regions. This method is preferred for clinical use because of short scan duration and computational simplicity, but is also subject to variability. Previous studies using [^11^C]PIB or [^18^F]AV45 have shown that cerebral blood flow changes in the target areas could induce apparent changes in SUVR [6,7]. This may be problematic for reliable assessment of longitudinal therapeutic response, as blood flow reductions up to 10% in the cortical target areas of AD patients have been reported over 3 years follow-up [6,8]. In addition, anti-amyloid drug therapy itself might change brain perfusion and thus tracer delivery [9,10]. Besides the effects of blood flow on [^18^F]AV45 SUVR, there is still major inconsistency regarding reference region selection for SUVR, thereby introducing an extra level of variability. The cerebellar grey matter (CB) has been the preferred reference region in clinical settings as it is notably free from fibrillar Aβ in sporadic AD. However, this region might be susceptible to perfusion deficits, decreased glucose metabolism, and Aβ deposition in late-stage dementia or early-onset familial AD [5,11–15]. Recent studies suggested using cerebral white matter (WM) as an alternative reference region for SUVR as SUV^WM^ did not significantly differ between diagnostic groups and its use as a reference resulted in reduced longitudinal variability and improved detection of cortical change [16–19]. Yet WM changes including demyelination, axonal disintegration or blood flow reduction during the course of AD might affect tracer uptake as well [15,20–22]. Blautzik et al. [23] recently suggested that WM normalization makes the SUVR more robust to changes in cerebral blood flow. However, this hypothesis should to be tested through a complete dynamic PET acquisition with tracer delivery rate K1 serving as a surrogate for blood flow [15,24]. Therefore to date, the influence of perfusion changes in the frequently chosen reference regions (CB, WM) on amyloid quantification by SUVR remains elusive.

The present study was designed to answer these aforementioned questions by simulating how [^18^F]AV45 SUVR is affected by cerebral blood flow changes. Both heterogeneous (target or reference region only) and global (combined target and reference region) changes were considered. Our study is novel compared to others [6,7] in several aspects: 1) we investigated the effect of a flow change in the CB or WM reference region and 2) arterial input functions and simulated time-activity curves (TACs) were based on our [^18^F]AV45 dynamic PET data acquired in a cohort of healthy control (HC), amnestic MCI (aMCI) and AD subjects [15].

A change in regional blood flow was simulated by varying K1 between -40 to +40% from its regional baseline value. The appropriate corresponding change in clearance rate k2 was considered whereas all other parameters (k3, k4, V_b_, arterial input function) were kept at their baseline values. In our simulations, the baseline values were either taken as the average HC values or as the measured values of the individual subject (including HC, aMCI and AD subjects). The subject-specific baseline allowed us to investigate whether flow effects vary between individual subjects or groups (e.g. HC vs AD).

## Materials and methods

### Subjects

Time activity curves were generated from kinetic parameters and input functions obtained from our previously described dynamic [^18^F]AV45 PET data [15]. The study included 10 patients with probable dementia due to AD, 15 patients with aMCI and 10 HCs. The demographics are summarised in Table 1. A panel of three board certified MDs experienced with neurodegenerative brain diseases and dementia made a consensus clinical diagnosis based on the NIA-AA diagnostic criteria [25]. Approval for the study was obtained from the Committee for Medical Ethics of the University of Antwerp / University Hospital Antwerp (14/12/130) and of Hospital Network Antwerp (ZNA) (4310). All procedures were in accordance with the Helsinki Declaration of 1975 and its later amendments or comparable ethical standards. All participants and/or their representatives provided written informed consent.

**Table 1:** Demographics.

**Parameter**	**HC (***n* **= 10)**	**aMCI (***n* **= 15)**	**AD (***n* **= 10)**
**Age (y)**	69 ± 6	74 ± 9	73 ± 5
**Sex (M|F)**	4|6	7|8	7|3
**Education (y)**	14 ± 2	11 ± 4	12 ± 4
**MMSE**	29 ± 1	25 ± 3 ^a^	22 ± 4 ^a^

Data are mean ± SD.

^a^Significantly different from controls (one-way ANOVA, corrected for multiple comparisons via Dunnett’s, p < 0.05).

Abbreviations: HC = healthy controls; aMCI = amnestic mild cognitive impairment; AD = Alzheimer’s disease; MMSE = Mini-Mental State Examination.

### Data acquisition and analysis

Details on PET protocol, radiometabolite analysis and MR imaging protocol are described elsewhere [15].

Briefly, [^18^F]AV45 was injected as an intravenous bolus of 288 ± 68 MBq and a 60 min dynamic PET scan (Siemens Biograph mCT TOF PET/CT) was acquired after injection. Continuous arterial blood sampling was performed simultaneously with the PET acquisition (Twilite, Swisstrace, Switzerland) to measure the radioactivity in whole blood. In addition, arterial blood samples were manually collected at discrete time points to determine the radioactivity in whole blood and plasma, as well as for determination of the plasma parent fraction by HPLC. For each subject, a 3-dimensional T1-weighted MRI scan (Siemens Trio Tim 3T MRI) was obtained.

Inter-frame motion correction of the PET images and PET-MRI co-registration were completed in PMOD v3.6 (PMOD Technologies Ltd., Zurich, Switzerland). The TACs were extracted from MR-based volumes-of-interest (VOIs) and corrected for partial volume effects. The bilateral VOIs included precuneus as a target region and cerebellar cortical grey matter (CB) and whole subcortical white matter (WM) as reference regions. Additional target regions were also investigated, including the four cortical lobes, poster cingulate and hippocampus. The 2-tissue 4-parameter compartment model with fitted blood volume fraction (2TCM-4k_V_b_) and metabolite-corrected plasma input function was applied to the regional TACs to determine the kinetic rate constants, including K1 (mL/min/mL) and k2 (1/min), the tracer transport rates from plasma to tissue and back, and k3 (1/min) and k4 (1/min), the tracer transport rates from the non-displaceable to the specific binding compartment and back, respectively. These kinetic parameters are used in our current simulations to build regional baseline TACs and derive regional baseline SUVRs.

### Simulations

Simulations were performed to assess how the SUVR measure is affected by both heterogeneous and global changes in blood flow. All simulations were performed using Matlab 2015b (The Mathworks, Natick, MA, USA).

#### Average HC baseline

Average HC baseline kinetic rate constants (i.e., K1, k2, k3, k4) and V_b_ of the precuneus, CB and WM were set to the average of the individual rate constants of all subjects in the HC group (Table 2) [15,26]. These HC baseline parameters were then used in combination with the average metabolite-corrected plasma input function to generate regional baseline TACs. The TACs were calculated as the convolution of the input function (C_plasma_(t)) with the tracer impulse response function (i.e., IRF(t;K1,k2,k3,k4)) in the tissue and a contribution from whole blood (C_b_(t)) [27]:

$$C_{tissue}(t)=C_{plasma}(t)\;⨂\;{IRF}_{tissue}(t;\;K1,k2,k3,k4)+C_{b}(t)\;V_{b}$$ (1)

**Table 2:** Average HC baseline [^18^F]AV45 kinetic rate constants and SUVR (at 50–60 min p.i.) used in the simulations.

**Baseline**	**V**_**b**_	**K1**	**k2**	**k3**	**k4**	**SUVR**^**prec**^
	(%)	(mL/min/mL)	(min^-1^)	(min^-1^)	(min^-1^)	
Precuneus	5.4 (6.4)	0.77 (4.9)	0.17 (4.2)	0.02 (18.5)	0.02 (14.7)	1.34 (ref CB); 0.55 (ref WM)
CB	5.2 (4.6)	0.66 (5.8)	0.17 (5.4)	0.01 (14.7)	0.02 (21.6)	
WM	2.0 (9.8)	0.28 (6.3)	0.14 (8.2)	0.19 (16.1)	0.04 (9.8)	

Values were obtained as the average values in the HC group from our previously reported human study [15]. Percentage standard error (SE) of parameters is denoted in parentheses.

Abbreviations: V_b_ = blood volume fraction; K1 = tracer delivery rate; SUVR = standardised uptake value ratio; ref CB = cerebellar grey matter reference region; ref WM = subcortical white matter reference region.

In the case of a 2-tissue compartment model, IRF is determined as K1•*f*(t;k2,k3,k4) = K1•[Θ_1_exp(-α_1_t)+Θ_2_exp(-α_2_t)] [27]. From the TACs, the SUVR_baseline_ was calculated as the ratio of tracer activity concentration in target and reference region (Table 2).

**Fig 1:**
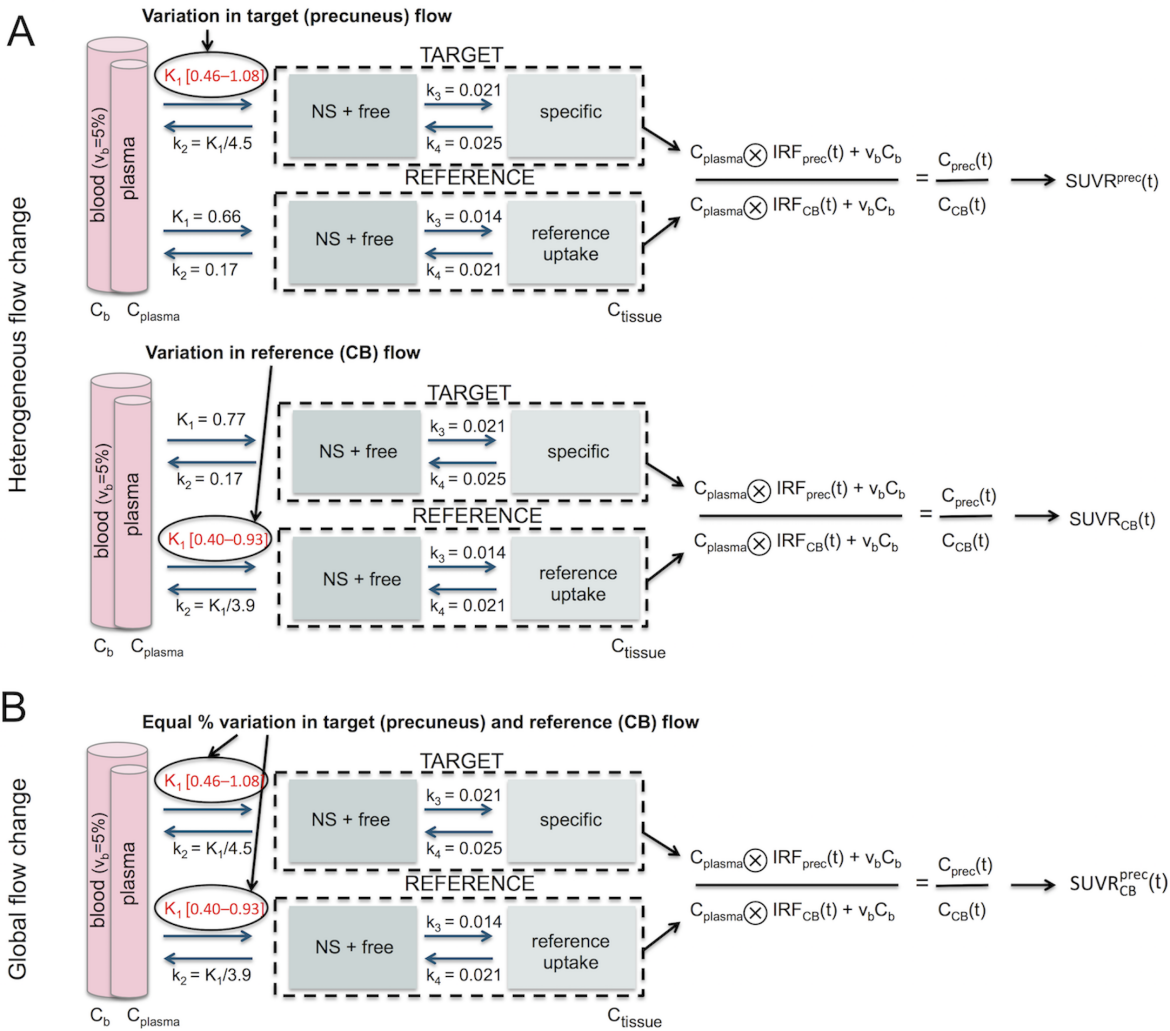
Simulation scheme based on the 2TCM describing the effect of a change in blood flow on SUVR for variable uptake periods. (A) Heterogeneous blood flow change: K1^prec^ (upper) or K1^CB^ (lower) was varied between [0.46–1.08] or [0.40–0.93] (corresponding to [-40%–+40%] from average HC baseline) with K1/k2 constant and all other parameters fixed to baseline values, to determine the corresponding SUVR^prec^ and SUVR_CB_, respectively. Remark that SUVR^prec^ and SUVR_CB_ are independent of the choice of the reference and target region, respectively. (B) Global blood flow change: K1^prec^ and K1^CB^ were simultaneously and in equal proportion varied to determine the corresponding ${\mathrm{SUVR}}_{\mathrm{CB}}^{\mathrm{prec}}$. In this figure, precuneus was chosen as the target and CB as the reference; simulations using different target or reference regions are similar. Abbreviations: NS = non-specific; C_plasma_ = metabolite-corrected plasma input function; IRF = 2TCM impulse response function; C_tissue_(t) = simulated time-activity curve in the tissue.

*Impact of simulated blood flow change on SUVR*. The simulation scheme to calculate the SUVR_simulated(K1,k2)_ for changing blood flow values is shown in Fig 1. Both heterogeneous (Panel A) and global (Panel B) blood flow changes were simulated.

*Heterogeneous blood flow change*. This simulation reflects a change in blood flow in the target region (e.g. precuneus) while keeping the blood flow in the reference region (either CB or WM) constant, or vice versa. A change in blood flow was simulated as a change in the tracer delivery rate K1 in the respective region. The K1 values were gradually increased or decreased from regional baseline value by 5, 10, 20 and 40%, the latter corresponding to the most extreme K1 values found in our entire cohort. Concurrently, the clearance rate k2 in that region was changed in order to keep the non-displaceable volume of distribution V_ND_ (K1/k2) constant. All other parameters were fixed at baseline values (see Table 2), and as a result the total volume of distribution V_T_ (= K1/k2(1+k3/k4)) was unaltered in the simulations. The blood flow-induced percentage change in SUVR of the target region was calculated as:

$$\Delta \mathrm{SUVR}=\left(\frac{{{\mathrm{SUVR}}_{\mathrm{simulated}(K1,k2)}\;\text{-}\;\mathrm{SUVR}}_{\mathrm{baseline}}}{{\mathrm{SUVR}}_{\mathrm{baseline}}}\right)\;100$$ (2)

and is denoted as ΔSUVR^target^ or ΔSUVR_ref_, depending on whether the blood flow was changed in target or reference region, respectively. Note that in the first case the percentage change ΔSUVR^target^ does not depend on the choice of reference region. In the second case, the change in ΔSUVR_ref_ does not depend on the target region (i.e., same percentage SUVR change in all possible target regions as long as the blood flow remains unaltered in these regions). Finally, the curve representing ΔSUVR at 50–60 min p.i. versus ΔK1 was generated and fitted with a third order polynomial for both cases. The used time interval was previously determined to be optimal for [^18^F]AV45 SUVR quantification [28].

*Global blood flow change*. This simulation reflects a simultaneous and equal blood flow change in the target and reference region (i.e. ΔK1^target^ = ΔK1^ref^). The corresponding percentage change in SUVR of the target region was calculated as:

$$\Delta {\mathrm{SUVR}}_{\mathrm{ref}}^{\mathrm{target}}=\left((1+\frac{{\Delta \mathrm{SUVR}}^{\mathrm{target}}}{100})(1+\frac{{\Delta \mathrm{SUVR}}_{\mathrm{ref}}}{100})\text{-}1\right)\;100$$ (3)

#### Subject-specific baseline

Apart from using the average kinetic parameters of the HC group as baseline and varying the K1, we also investigated the effect of a K1 variation on the SUVR of each individual HC, aMCI and AD subject. To this end, regional baseline TACs were built for each subject separately using the subject’s specific kinetic parameters and metabolite-corrected plasma input function. Table 2 in [15] reports the average kinetic parameters (± SD) over the three diagnostic groups. For each subject, blood flow was increased or decreased by 5, 10, 20 and 40% from its own baseline K1 value and the corresponding ΔSUVR was calculated as described previously. The individual ΔSUVR vs ΔK1 regression curves were then averaged per diagnostic group for reporting.

### Statistical analysis

Analysis of variance (2-way ANOVA) with post-hoc Tukey’s multiple comparisons test was used to detect the differences in the average ΔSUVR vs ΔK1 curves of the three diagnostic groups. The dependent variable was ΔSUVR while the independent variables were ΔK1 and diagnostic group. The interaction between diagnostic group and ΔK1 was evaluated. Statistical analysis was performed in JMP Pro v12 (SAS Institute Inc., USA).

## Results

There was no significant difference in age, sex or years of education between all groups (Table 1). One AD subject was excluded because the precuneus could not be properly delineated on the MRI, leaving 9 AD, 15 aMCI and 10 HC subjects for analysis.

### Average HC baseline: Flow-induced SUVR changes

#### Heterogeneous blood flow change

**Fig 2:**
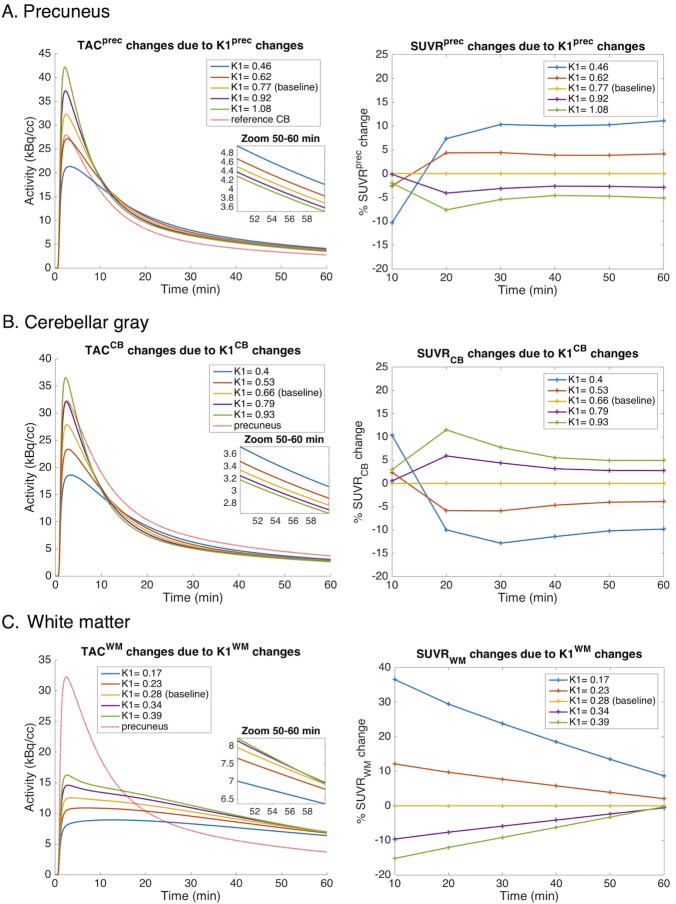
Impact of simulated blood flow change on regional activity concentration and SUVR as a function of uptake time. (left) Simulated TACs of the precuneus (A), CB (B), and WM (C) for various K1^prec^, K1^CB^, and K1^WM^, respectively. (right) Percentage change in SUVR^prec^ (A), SUVR_CB_ (B), and SUVR_WM_ (C) as a function of time for various K1^prec^, K1^CB^, and K1^WM^, respectively. Simulated K1 changes corresponded to -40, -20, +20, and +40% relative to average HC baseline. Remark that % SUVR^prec^ and % SUVR_CB or WM_ change are independent of the choice of the reference and target region, respectively. Abbreviations: TAC = time-activity curve; K1 = tracer delivery rate; SUVR = standardised uptake value ratio; prec = precuneus; CB = cerebellar grey matter; WM = subcortical white matter.

Flow changes altered the regional TACs (Fig 2 left) and SUVRs (Fig 2 right). The amplitude and direction of change depended on the region, the direction of blood flow change relative to baseline, and the tracer uptake period. Decreasing K1^prec^ (Fig 2A) or K1^CB^ (Fig 2B) increased TAC^prec^ or TAC^CB^, respectively, at later time points. As a result, there was an increased SUV^prec^ or SUV^CB^ leading to an increased SUVR^prec^ or decreased SUVR_CB_, respectively. Note that the change in SUVR^prec^ and SUVR_CB_ do not depend on the choice of the reference region and target region, respectively. Decreasing K1^WM^ (Fig 2C) decreased TAC^WM^ at later time points and correspondingly decreased SUV^WM^ and increased SUVR_WM_. When K1^WM^ was hypothetically increased by more than 40% (i.e. K1^WM^ > 0.4 mL/min/mL) during the simulation, the TAC^WM^ again decreased, corresponding to a decreasing SUV^WM^ (data not shown).

**Fig 3:**
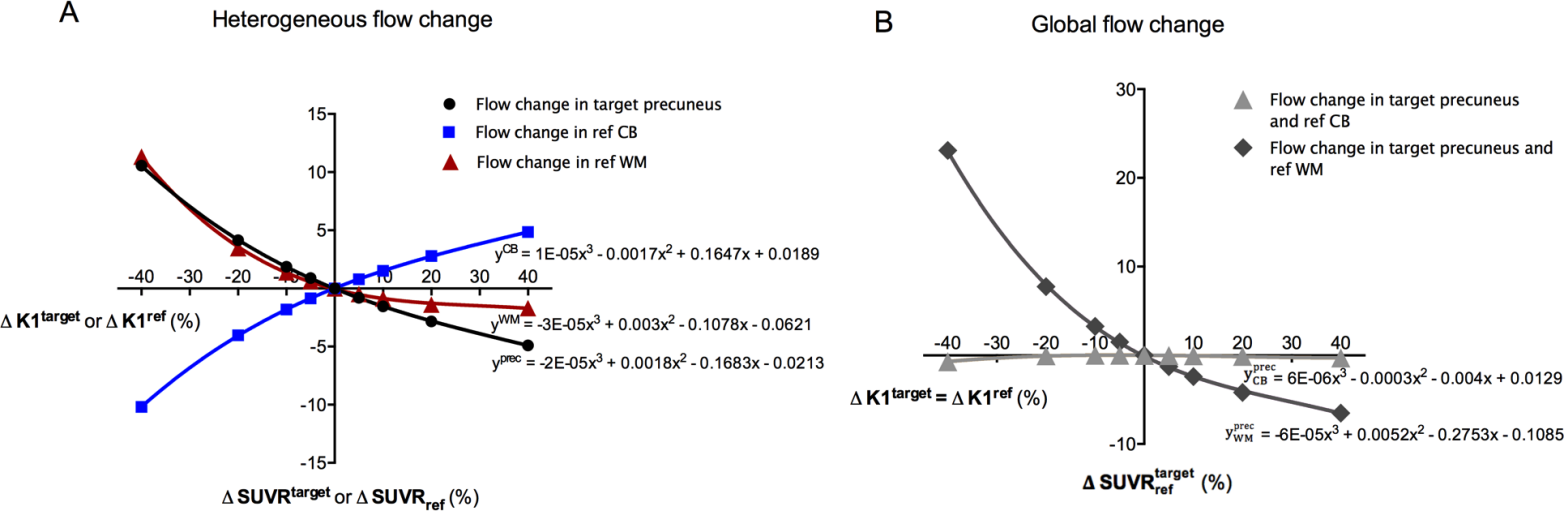
Regression curves of ΔSUVR (50–60 min p.i.) versus ΔK1 derived from simulations. (A) Heterogeneous blood flow changes: ΔSUVR^prec^, ΔSUVR_CB_, or ΔSUVR_WM_ versus ΔK1^prec^ (black), ΔK1^CB^, (blue) or ΔK1^WM^ (red), respectively. Remark that ΔSUVR^prec^ and ΔSUVR_CB or WM_ are independent of the choice of the reference and target region, respectively. (B) Homogeneous blood flow changes: $\Delta {\mathrm{SUVR}}_{\mathrm{CB}}^{\mathrm{prec}}$ or $\Delta {\mathrm{SUVR}}_{\mathrm{WM}}^{\mathrm{prec}}$ versus ΔK1^prec^ = ΔK1^CB^ (light grey) or ΔK1^prec^ = ΔK1^WM^ (dark grey), respectively. Simulated K1 changes corresponded to -40, -20, -10, -5, +5, +10, +20, and +40% relative to average HC baseline. A third order polynomial function was fitted to the curves (r^2^ = 0.99). Abbreviations: K1 = tracer delivery rate; SUVR = standardised uptake value ratio; prec = precuneus; CB = cerebellar grey matter; WM = subcortical white matter.

The effect of heterogeneous blood flow variations on SUVR at the 50 to 60 min p.i. interval is summarised in Fig 3A. When K1^prec^, K1^CB^, or K1^WM^ were varied from -40 to +40% relative to HC baseline, the corresponding SUVR^prec^, SUVR_CB_, or SUVR_WM_ changed from +10.6 to -4.9%, from -10.2 to +4.8%, or from +11.3 to -1.7%, respectively.

#### Global blood flow change

The effect of global blood flow variations is shown in Fig 3B. When K1^prec^ and K1^CB^ were simultaneously and equally varied from -40% to +40% relative to HC baseline, the corresponding ${\mathrm{SUVR}}_{\mathrm{CB}}^{\mathrm{prec}}$ changed from -0.7 to -0.3% whereas for K1^prec^ and K1^WM^ the ${\mathrm{SUVR}}_{\mathrm{WM}}^{\mathrm{prec}}$ variation was from +23.1 to -6.5%.

#### Other target regions

Table 3 reports simulated ΔK1^target^ and corresponding ΔSUVR^target^ values for other target regions, including the four cortical lobes, poster cingulate and hippocampus. Blood flow effects were highest in the hippocampus and lowest in the poster cingulate. There was a significant inverse relationship between ΔSUVR^target^ and V_T_ of the target region (Pearson’s r: -0.96, p = 0.0008).

**Table 3:** ΔSUVR^target^ in alternative target areas due to simulated ΔK1^target^.

**Target area**	**ΔK1**^**target**^**: -10%**	**ΔK1**^**target**^**: -30%**
Frontal lobe	+1.7	+6.6
Parietal lobe	+1.5	+5.8
Temporal lobe	+1.7	+6.2
Occipital lobe	+1.3	+4.9
Precuneus	+1.9	+7.2
Poster cingulate	+0.9	+3.0
Hippocampus	+2.8	+9.8

ΔSUVR^target^ (%) in alternative target areas due to simulated blood flow reductions of -10 or -30% compared to average HC baseline in these areas.

### Subject-specific baseline: Flow-induced SUVR changes

In each HC, aMCI, or AD subject, we evaluated the effect of a regional flow variation on its individual SUVR value and calculated the corresponding ΔSUVR.

#### Heterogeneous blood flow change

**Fig 4:**
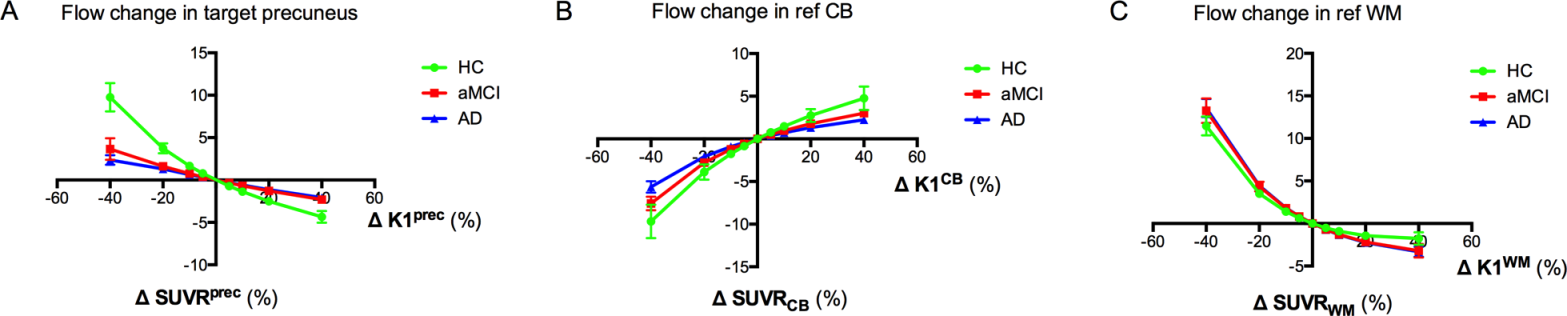
Regression curves for each diagnostic group based on subject-specific baseline and a heterogeneous blood flow change. The subject-specific curves of ΔSUVR^prec^ (A), ΔSUVR_CB_ (B) or ΔSUVR_WM_ (C) versus ΔK1^prec^, ΔK1^CB^, or ΔK1^WM^, respectively, were averaged per diagnostic group (average ± SD). There was a significant difference between HC and aMCI (p = 0.012, ANOVA) and AD (p = 0.005) for the precuneus. Remark that ΔSUVR^prec^ and ΔSUVR_CB or WM_ are independent of the choice of the reference and target region, respectively. Abbreviations: K1 = tracer delivery rate; SUVR = standardised uptake value ratio; prec = precuneus; CB = cerebellar grey matter; WM = subcortical white matter.

For a heterogeneous blood flow change in the precuneus target region, the corresponding SUVR^prec^ change was significantly higher in HC compared to aMCI (p = 0.012) and AD (p = 0.005) (Fig 4). No significant difference between the groups was found when blood flow was varied in the reference region. Note that the average curves of the HC subjects (green colour) coincide with those of Fig 3 for all regions, as expected. In the AD group, when K1^prec^, K1^CB^, or K1^WM^ were varied from -40 to +40% relative to the subject-specific baseline, the corresponding SUVR^prec^, SUVR_CB_, or SUVR_WM_ changed on average from +2.5±2 to -2.0±1%, from -5.5±2 to +2±1%, or from +13.5±3 to -3.5±2%, respectively. In AD, the ΔSUVR^prec^ versus ΔK1^prec^ curve could be approximated by a linear function (y = -0.057x+0.058, r^2^ = 0.99).

#### Global blood flow change

**Fig 5:**
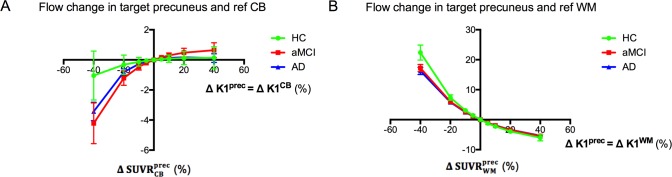
Regression curves for each diagnostic group based on subject-specific baseline and a global blood flow change. The subject-specific curves of $\Delta {\mathrm{SUVR}}_{\mathrm{CB}}^{\mathrm{prec}}$ (A) or $\Delta {\mathrm{SUVR}}_{\mathrm{WM}}^{\mathrm{prec}}$ (B) versus ΔK1^prec^ = ΔK1^CB^ or ΔK1^prec^ = ΔK1^WM^, respectively, were averaged per diagnostic group (average ± SD). There was a significant difference between HC and aMCI (p = 0.024, ANOVA) and AD (p = 0.018) for the precuneus and reference WM. Remark that ΔSUVR^prec^ and ΔSUVR_CB or WM_ are independent of the choice of the reference and target region, respectively. Abbreviations: K1 = tracer delivery rate; SUVR = standardised uptake value ratio; prec = precuneus; CB = cerebellar grey matter; WM = subcortical white matter.

For a global flow change, the corresponding ${\mathrm{SUVR}}_{\mathrm{WM}}^{\mathrm{prec}}$ change was significantly higher in HC compared to aMCI (p = 0.024) and AD (p = 0.018) (Fig 5). No significant differences were found for reference CB. In the AD group, when K1^prec^ and K1^CB^ were simultaneously and equally varied from -40% to +40% relative to the subject-specific baseline, the corresponding ${\mathrm{SUVR}}_{\mathrm{CB}}^{\mathrm{prec}}$ changed on average from -3.5±2 to +0.1±1%, whereas for K1^prec^ and K1^WM^ the ${\mathrm{SUVR}}_{\mathrm{WM}}^{\mathrm{prec}}$ variation was from +16.0±4 to -5.5±2%.

## Discussion

This study reports on the influence of cerebral blood flow changes on the quasi-steady-state [^18^F]AV45 SUVR measure for Aβ plaque load.

We found that blood flow alterations in two frequently chosen reference regions exerted opposite effects on their respective SUVRs of the target region: a blood flow decrease in WM caused an increase in SUVR_WM_ (due to a decreased SUV of WM) whereas a blood flow decrease in CB caused a decrease in SUVR_CB_ (due to an increased SUV of CB), irrespective of the chosen target region. Lowering the blood flow in the precuneus grey matter increased SUVR^prec^ (due to an increased SUV of precuneus), irrespective of the chosen reference region. These observations might be explained by significantly slower delivery (i.e., K1) and clearance (i.e., k2) in white matter regions compared to grey matter regions, in combination with a significantly higher k3 of white matter (with k3 > k2). Indeed, if K1^WM^ was increased or k3^WM^ was decreased towards the grey matter range (i.e., K1^WM^ > 0.4 mL/min/mL, k3^WM^ < 0.10 min^-1^), the flow-effect on SUV^WM^ changed in the direction similar to that of the grey matter regions (i.e., lower flow results in higher SUV; data not shown). The slower clearance of [^18^F]AV45 from white matter tissue together with the lipophilic nature of [^18^F]AV45 might contribute to the high WM binding reported for [^18^F]AV45, assumed as being largely non-specific (e.g., binding to the β-sheet structure of myelin) [29,30]. The precuneus (medial parietal cortex) was chosen as the main target region of interest in this study, because this region is known to exhibit early alterations in cerebral blood flow and Aβ deposition during the course of Alzheimer’s disease. Similar blood flow effects were found in other cortical grey matter regions.

A heterogeneous blood flow reduction due to reduced flow in the target area compared with the reference area (i.e., reduced relative delivery rate R1) is a likely phenomenon during prodromal or early AD [31]. Quantification of this flow effect on SUVR has been previously investigated by van Berckel et al. for [^11^C]PIB [6] where a -40% reduction of R1 relative to baseline (R1 = 1) induced less than 5% increase in the SUVR of a typical AD region at 50–60 min p.i. This is in line with our results, as a decrease of -40% in R1 due to a decrease of -40% in K1^target^ (or analogous but less likely, an increase in K1^CB^) caused less than 5% increase in the corresponding SUVR. In addition to heterogeneous blood flow changes, global blood flow changes are likely to occur in AD due to, for example, daily variations in cerebral blood flow or administration of drugs [9,10,32]. For a global blood flow change of -40% and CB normalization, van Berckel et al. showed approximately a -10% reduction of SUVR. This is slightly higher than the -3.5% reported here for the AD group. This discrepancies between the two studies could be attributed to tracer differences ([^11^C]PIB with a 60% stronger affinity compared to [^18^F]AV45 [33]), the lower K1 range for a typical grey matter region (< 0.44 mL/min/mL in [6], compared to > 0.4 mL/min/mL in our study) and the choice of baseline. Cselenyi et al.[7] also reported the inverse relationship between R1 and SUVR based on single-occasion experimental [^18^F]AV45 data in HC, MCI, and early AD. However, none of these studies [6,7] have explored changes in R1 due to flow variation in the reference region CB or WM, while keeping the flow in the target regions constant.

We found that a global blood flow change (equal changes in both the target and reference) caused a limited change in ${\mathrm{SUVR}}_{\mathrm{CB}}^{\mathrm{target}}$, while the effect was larger for ${\mathrm{SUVR}}_{\mathrm{WM}}^{\mathrm{target}}$. This is due to the fact that a blood flow reduction in the white matter reinforced the effect of a blood flow reduction in the target region whereas blood flow reductions in cerebellar grey matter opposed this effect. We conclude that WM normalization makes the semi-quantitative SUVR measure more susceptible to changes in global blood flow than CB normalization.

Several recent longitudinal amyloid PET studies have reported that WM normalization results in improved quantitative stability of SUVR compared to CB normalization, either due to biological or scanner-related physical effects [16–19]. Blautzik et al. [23] hypothesised that this might be due to ${\mathrm{SUVR}}_{\mathrm{WM}}^{\mathrm{target}}$ being more robust to changes in cerebral blood flow. The authors suggested the existence of a functional coupling and common vascular supply between WM and neocortical target areas. The current study demonstrated that increasing target SUVs due to a global reduction in perfusion are not counterbalanced by increasing SUVs in the WM, but on the contrary are reinforced by decreasing WM SUVs. Therefore, WM normalization should be applied with caution in (therapy) monitoring studies with elevated (or reduced) global blood flow, as the false positive (or negative) rate may increase. Previously, our group reported that ${\mathrm{SUVR}}_{\mathrm{CB}}^{\mathrm{target}}$ was paradoxically decreased from the aMCI towards the AD stage, whereas both ${\mathrm{SUVR}}_{\mathrm{WM}}^{\mathrm{target}}$ and V_T_ were increased [15]. At first glance, this underestimation of the difference in Aβ between both groups by ${\mathrm{SUVR}}_{\mathrm{CB}}^{\mathrm{target}}$ could be explained by the increased ${R1}_{\mathrm{CB}}^{\mathrm{target}}$ in AD compared to aMCI, which might be related to prominent cerebellar blood flow reductions at late-stage AD (e.g., due to cerebellar degeneration and cerebellar diaschisis effects from remote affected cortical regions [11]). However, flow effects were too small and could only partially explain these observations.

The current study also evaluated the effect of the subject’s specific kinetics. When grouped per diagnosis the magnitude of a flow-induced SUVR^prec^ change was found to be twice as high for the HC subjects than for the AD subjects. Similarly, in the case of a global blood flow change, the magnitude of the blood flow effect was different between the HC and AD subjects when SUVR was referenced to the WM, with changes up to +23% in HC versus +16% in AD (for comparison, -1% versus -3.5% when considering the CB reference). As Aβ-modifying drug trials are moving towards preclinical disease stages [34], one should therefore carefully consider that the flow-induced effects on SUVR are likely more prominent in the presence of less cortical Aβ load. This was in line with low Aβ-binding regions (e.g., hippocampus) showing higher flow-induced SUVR changes compared to the high Aβ-binding regions (e.g., posterior cingulate), both in HC and AD.

A limitation of this study is the choice of K1 ranges, which are based on a cross-sectional dataset rather than longitudinal flow and SUVR measures within the same subject. Therefore, blood flow changes were simulated by varying K1 over a large but biological acceptable range based on our dataset. Since up to 84% of aged subjects show morphological substrates of cerebrovascular disease in addition to AD pathology [35], we assume that the range in our dataset [15] also covers the worst-case scenarios. We thus may conclude that even in the presence of vascular deficits, artificial blood flow-induced changes in SUVR remain in a relatively small range, especially when CB normalization is applied. While our findings have limited effect in a diagnostic setting (i.e., to determine Aβ positivity or negativity) we suggest to take blood flow effects into account for reliable assessment of follow-up PET scans and longitudinal Aβ-modifying treatment effects.

## Conclusion

This study addressed blood flow deficits in target and reference regions as a possible source of bias in the quantification of cortical Aβ load by [^18^F]AV45 SUVR. Flow reductions in the target region caused an increased SUVR of the target, whereas flow reductions in the references CB and WM resulted in a decreased and increased SUVR of the target, respectively. It was concluded that artificial blood flow-induced changes in SUVR remain in a relatively small range. However with regard to possible treatment-induced global flow variations, WM normalization should be applied with caution. Lastly, the magnitude of a simulated flow effect varies between diagnostic groups and was more prominent in cortical target areas of HC compared to aMCI or AD. This was in line with low Aβ-binding regions showing higher flow-induced SUVR changes compared to high Aβ-binding regions for all diagnostic groups.
